# Statin and the risk of hepatocellular carcinoma in patients with hepatitis B virus or hepatitis C virus infection: a meta-analysis

**DOI:** 10.1186/s12876-020-01222-1

**Published:** 2020-04-09

**Authors:** Xiaofei Li, Lina Sheng, Liwen Liu, Yongtao Hu, Yongxin Chen, Lianqing Lou

**Affiliations:** Department of infectious diseases, Yiwu Central Hospital, No. 519 Nanmen Street, Yiwu, 322000 China

**Keywords:** Statin, Hepatocellular carcinoma, Hepatitis B virus, Hepatitis C virus, Cirrhosis, Meta-analysis

## Abstract

**Background:**

Statin may confer anticancer effect. However, the association between statin and risk of hepatocellular carcinoma (HCC) in patients with hepatitis B virus (HBV) or hepatitis C (HCV) virus infection remains inconsistent according to results of previous studies. A meta-analysis was performed to summarize current evidence.

**Methods:**

Related follow-up studies were obtained by systematic search of PubMed, Cochrane’s Library, and Embase databases. A random-effect model was used to for the meta-analysis. Stratified analyses were performed to evaluate the influences of study characteristics on the outcome.

**Results:**

Thirteen studies with 519,707 patients were included. Statin use was associated with reduced risk of HCC in these patients (risk ratio [RR]: 0.54, 95% CI: 0.44 to 0.66, *p* < 0.001; I^2^ = 86%). Stratified analyses showed that the association between statin use and reduced HCC risk was consistent in patients with HBV or HCV infection, in elder (≥ 50 years) or younger (< 50 years) patients, in males or females, in diabetic or non-diabetic, and in those with or without cirrhosis (all *p* < 0.05). Moreover, lipophilic statins was associated with a reduced HCC risk (RR: 0.52, *p* < 0.001), but not for hydrophilic statins (RR: 0.89, *p* = 0.21). The association was more remarkable in patients with highest statin accumulative dose compared to those with lowest accumulative dose (*p* = 0.002).

**Conclusions:**

Satin use was independently associated with a reduced risk of HCC in patients with HBV or HCV infection.

## Background

Hepatocellular carcinoma (HCC) is one of the most prevalent cancers in digestive system, and approximately 500,000 cases of HCC are newly diagnosed annually worldwide [1]. Patients with HCC are of poor prognosis due to limited treatment options, and the median survival of these patients is less than 1 year [2–4]. Patients with hepatitis B virus (HBV) or hepatitis C virus (HCV) infection are primarily the high-risk population for the development of HCC [2–4]. Although HBV suppression or HCV eradication has been increasingly applied, the incidence of HCC in patients with HBV or HCV infection remains high [5, 6]. Therefore, identification of novel chemoprevention agents for HCC remains of great clinical importance, particularly for high-risk population such as patients with HBV or HCV infection [7].

Statins, also known as 3-hydroxy-3-methylglutaryl CoA (HMG-CoA) reductase inhibitors, are a category of cholesterol-lowering medications which have become the mainstays for the primary and secondary prevention of cardiovascular diseases [8]. Moreover, increasing evidence demonstrates the potential pleiotropy of statins, such as anti-inflammation, immunomodulation, pro-apoptosis, anti-proliferation, and anti-invasion, all of which have been implicated in carcinogenesis and metastasis [9, 10]. Therefore, statins have been suggested as anticancer agents [11]. A previous meta-analysis indicated that use of statin may be related with a 37% reduced risk of HCC incidence [12]. However, this meta-analysis included a patient population of heterogeneous spectrum of clinical statuses, which makes the interpretation of the results difficult [12]. Moreover, cross-sectional studies were included despite of follow-up studies, which may introduce additional biases [12]. Besides, previous studies evaluating the association between statin use and HCC risk in patients with HBV or HCV infection retrieved inconsistent results. Although most studies indicated that statin use was associated with a reduced risk of HCC in patients with HBV or HCV infection [13–22], some studies showed a nonsignificant association between statin use and HCC risk in these patients [23–25]. Therefore, we aimed to perform a meta-analysis of longitudinal follow-up studies to systematically evaluate the association between statin use and HCC risk in high-risk patients with HBV and HCV infection. Moreover, we explored the potential influences of study characteristics on this association, including virus type, age, gender, diabetic status of the patients, with or without cirrhosis, characteristics of statins, and accumulative dose of statins.

## Methods

The MOOSE (Meta-analysis of Observational Studies in Epidemiology) [26] and Cochrane’s Handbook [27] guidelines were followed during the designing, performing, and reporting of the meta-analysis.

### Literature search

Systematic search of electronic databases of PubMed, Cochrane’s Library, and Embase were performed to identify potentially relevant studies, via the following terms: (1) “statin” OR “3-hydroxy-3-methyl-glutaryl CoA reductase inhibitor” OR “CS-514” OR “statin” OR “simvastatin” OR “atorvastatin” OR “fluvastatin” OR “lovastatin” OR “rosuvastatin” OR “pravastatin” OR “pitavastatin”; and (2) “chronic hepatitis B” OR “chronic hepatitis C” OR “hepatitis B virus” OR “hepatitis C virus” OR “HBV” OR “HCV”. We used this extensive search strategy to avoid missing of potentially relevant studies. The search was limited to human studies, and no language restriction was applied. Besides, we also studied the reference lists of related original studies and review articles using a manual approach. The final literature search was conducted on September 15, 2019.

### Study selection

The inclusion criteria were: (1) full-length articles reporting longitudinal follow-up studies, including randomized controlled trials (RCTs), cohort studies, and nested case-control studies; (2) enrolled at least 1000 adult patients with HBV or HCV infection and without HCC at baseline; (3) investigated the association between statin use and HCC risk during follow-up, with a minimal follow-up duration of 1 year; and (4) reported the relative risk for this association after adjustment of potential confounding factors. Review articles, preclinical studies, and studies irrelevant to the purpose of current meta-analysis were excluded.

### Data extracting and quality evaluation

Two authors indepdently performed database search, data extraction, and study quality assessment according to predefined criteria. If discrepancies occurred, they were solved by consensus between the two authors or discussion with the corresponding author. Data extracted included: (1) study information: name of first author, publication year, and study country; (2) study design characteristics; (3) patient characteristics: disease status, sample size, age, sex, prevalence of diabetes, and proportions of patients with cirrhosis at baseline; (4) definition of statin use; (5) follow-up durations; (6) strategy for HCC validation and number of HCC cases during follow-up; and (7) confounding factors adjusted. The Newcastle-Ottawa Scale was used as an instrument for study quality evaluation [28]. This scale ranges from 1 to 9 stars, and assesses study quality mainly regarding three domains, including study group selection, between-group comparability, and validation of the outcome of interest.

### Statistical analyses

A risk ratio (RR) with corresponding 95% confidence interval (CI) was used as the main measure for the association between statin use and HCC risk in patients with HBV or HCV infection. Data of RRs and their corresponding stand errors (SEs) were calculated from 95% CIs or *p* values, and a logarithmical transformation was performed to stabilize variance and normalized the distribution [27]. The Cochrane’s Q test was performed to evaluate the heterogeneity, and the I^2^ statistic was also estimated [29]. An I^2^ > 50% indicates significant heterogeneity. We used a random-effect model for the meta-analysis of RR data because this model incorporates the potential heterogeneity among the included studies to calculate a more generalized result [27], By omitting one individual study at a time, we performed sensitivity analyses to test the robustness of the results [30]. We also performed stratified analyses to evaluate the influences of virus type, age, gender, diabetic status, with or without cirrhosis, lipophilic or hydrophilic statins, and accumulative dose of statin on the results. The potential publication bias was initially detected by visual inspection of the symmetry of funnel plots, then complemented with the Egger’s regression asymmetry test [31]. RevMan (Version 5.1; Cochrane Collaboration, Oxford, UK) software was used for the meta-analysis.

## Results

### Literature search

**Fig. 1:**
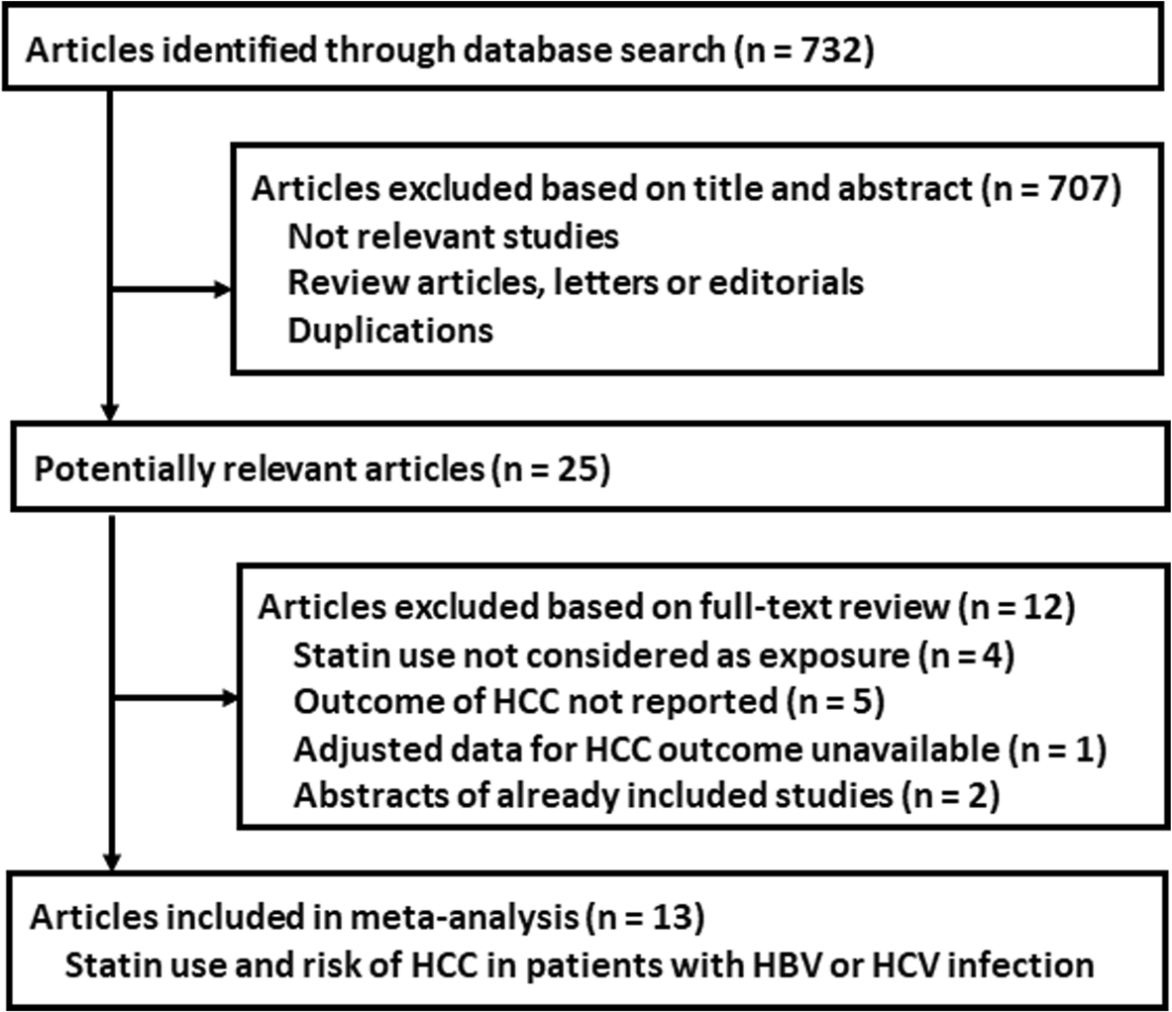
Flowchart of database search and study identification

Figure 1 shows the literature search process. Briefly, 732 articles were obtained via initial search of the PubMed, Cochrane’s Library, and Embase databases, and 707 were excluded through screening of the titles and abstracts mainly because they were not relevant to the purpose of the meta-analysis. Subsequently, 25 records underwent full-text review. Of these, 12 were further excluded because four of them did not evaluate statin use as exposure, five did not report outcome of HCC risk, one did not provide available data for the multivariate adjusted association between statin use and HCC risk, and the remaining three were abstracts of already included studies. Finally, we included 13 studies in this meta-analysis [13–25].

### Study characteristics and quality evaluation

The characteristics of the studies were presented in Table 1. All of them were observational studies, among which one was a prospective cohort study, another one was a nested case-control study, and the remaining 11 were retrospective cohort studies. Since two studies reported the association between statin use and HCC risk in patients with HBV and HCV infection separately, these datasets were included independently [22, 24]. Overall, 15 datasets from 13 studies, with 519,707 adult patients with HBV or HCV infection were included [13–25]. These studies were performed in China [13, 14, 16, 17, 21, 24], Korea [19, 20], the US [15, 18, 23, 25], and Sweden [22]. The mean age of the patients varied from 35 to 64 years, with percentiles of male ranging from 49 to 98%. Statin use was validated by prescription records in all studies and defined by accumulative statin dose of more than 28~30 cumulative defined daily dose (cDDD) in most studies [13–17, 20–23]. The follow-up duration varied from 2.5 to 10.7 years. The International Classification of Diseases (ICD) version 9 or 10 codes were used to validate HCC cases, and a total of 40,588 patients with HCC were included. Potential confounding factors including age, sex, diabetic status, comorbidities, and concurrent medications, were adjusted when presenting the outcome. The NOS scores of the included studies ranged from seven to nine, indicating generally good study quality.

**Table 1:** Characteristics of the included follow-up studies

Study	Country	Design	Patient characteristics	Sample size	Mean age years	Male %	Diabetes %	Cirrhosis %	Validation of statin use	Follow-up durations years	HCC validation	HCC cases	Variables adjusted	NOS
Tsan 2012 [13]	China	RC	Adult patients with a first-time diagnosis of HBV infection	33413	35.6	58.2	26.4	10.7	Records of prescription for statins ≥28 cDDD	9.8	ICD-9 codes	1021	Age, sex, income, urbanization, diabetes, and liver cirrhosis	9 (4, 2, 3)
Tsan 2013 [14]	China	RC	Adult patients with a first-time diagnosis of HCV infection	260864	50.4	49.2	27.6	18.4	Records of prescription for statins ≥28 cDDD	10.7	ICD-9 codes	27883	Age, sex, income, urbanization, diabetes, and liver cirrhosis	9 (4, 2, 3)
Butt 2015 [15]	the US	RC	Adult patients with HCV infection	7248	52.4	95.5	13.1	0	Records of prescription for statins ≥28 cDDD	10	ICD-9 codes	142	Age, race, sex, development of cirrhosis, HCV RNA, BMI, dyslipidemia, diabetes, and alcohol abuse	8 (3, 2, 3)
Hsiang 2015 [17]	China	RC	Adult patients with HBV infection	53513	58.9	66.8	45.4	3.1	Records of prescription for statins ≥28 cDDD	4.6	ICD-9 codes	6883	Age, sex, cirrhosis and complications, and diabetes	8 (3, 2, 3)
Chen 2015 [16]	China	RC	Adult patients with HBV infection	71847	41.5	57.8	NR	NR	Records of prescription for statins ≥28 cDDD	9	ICD-9 codes	1735	Age, sex. CCI index, and using of other medications	7 (2, 3, 2)
Mohanty 2016 [18]	the US	RC	Adult patients with HCV related compensated cirrhosis	40512	56	98	34.1	100	Records of prescription for statins ≥2 fills	2.5	ICD-9 codes	173	Age, race, sex, BMI, dyslipidemia, diabetes, and MELD Score	7 (2, 3, 2)
Simon 2016 [23]	the US	RC	Adult patients with HCV infection without cirrhosis	9135	52.9	95.7	17.1	0	Records of prescription for statins ≥28 cDDD	7.4	ICD-9 codes	239	Age, sex, race, smoking, alcohol abuse, BMI, diabetes, and concurrent medications	8 (3, 2, 3)
Chang 2017 [24]	China	RC	Adult patients with HBV or HCV related cirrhosis	1350	57	73	74.5	100	Records of prescription for statins ≥28 cDDD	5.5	ICD-9 codes	111	Age, sex, CCI index, diabetes, and concurrent medications	7 (2, 3, 2)
Kim 2017 [19]	Korea	NCC	Adult patients with HBV or HCV infection	1374	52.5	81.4	100	8.9	Records of prescription for statins	5	ICD-10 codes	229	Age, sex, diabetic duration, CCI, and concurrent medications	7 (2, 3, 2)
Simon 2019 [22]	Sweden	PC	Adult patients with HBV or HCV infection	16668	47.4	65.5	30.5	10	Records of prescription for statins ≥30 cDDD	8	ICD-10 codes	616	Age, sex, cirrhosis and complications, diabetes, and concurrent medications	8 (3, 2, 3)
Kaplan 2019 [25]	the US	RC	Adult patients with HCV infection	5455	64	97.6	70.8	100	Records of prescription for statins	8	ICD-10 codes	133	Age, sex, CCI, diabetes, comorbidities, and concurrent medications	7 (2, 3, 2)
Goh 2019 [20]	Korea	RC	Adult patients with HBV infection	7713	47.3	66.2	11.4	24.1	Records of prescription for statins ≥28 cDDD	9.2	ICD-10 codes	702	Age, sex, cirrhosis, diabetes, hypertension, HBV DNA level, antiviral treatment, and antiplatelet therapy	9 (4, 2, 3)
Lee 2019 [21]	China	RC	Adult patients with HBV infection	10615	58.8	72.4	29	17.1	Records of prescription for statins ≥28 cDDD	5	ICD-10 codes	721	Age, sex, cirrhosis, diabetes, hypertension, hyperlipidemia, and concurrent medications	8 (3, 2, 3)

*HCC* hepatocellular carcinoma, *NOS* the Newcastle-Ottawa Scale, *RC* retrospective cohort, *PC* prospective cohort, *NCC* nested case-control, *HBV* hepatitis B virus, *HCV* hepatitis C virus, *cDDD* cumulative defined daily dose, *ICD* International Classification of Diseases, *BMI* body mass index, *CCI* Charlson comorbidity index, *MELD* model for end-stage liver disease

### Results of main meta-analysis

**Fig. 2:**
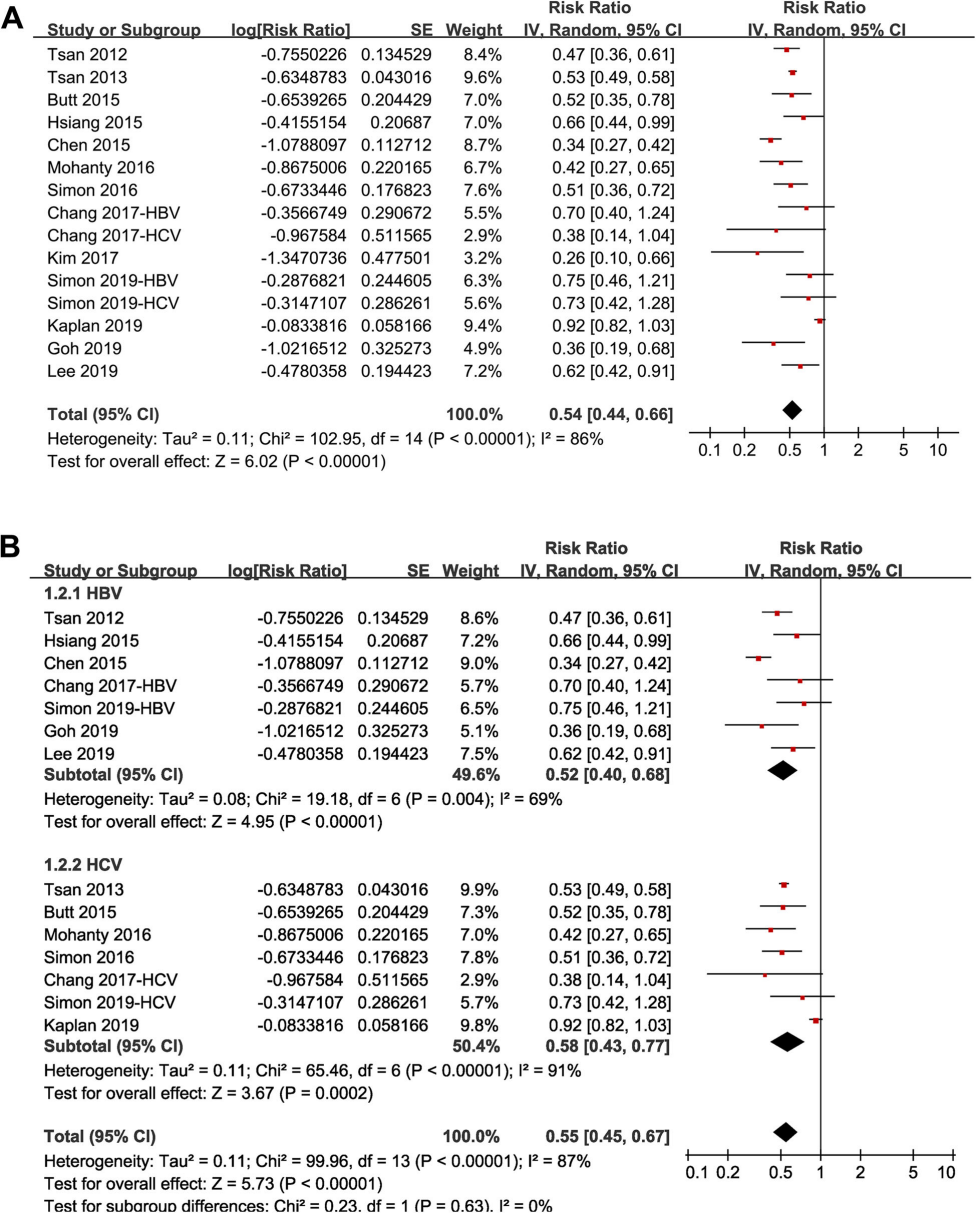
Forest plots for the meta-analysis of the association between statin use and HCC risk in patients with HBV or HCV infection: **a** overall meta-analysis; and **b** stratified analyses according to type of virus infected

Pooled results of all included studies using a random-effect model showed that statin use was associated with a reduced risk of HCC in patients with HBV or HCV infection (RR: 0.54, 95% CI: 0.44 to 0.66, *p* < 0.001; Fig. 2a) with significant heterogeneity (p for Cochrane’s Q test < 0.001, I^2^ = 86%). Sensitivity analyses by omitting one datasets at a time did not significantly change the results (RR: 0.50 to 0.56, p all < 0.05).

### Results of stratified analyses

**Fig. 3:**
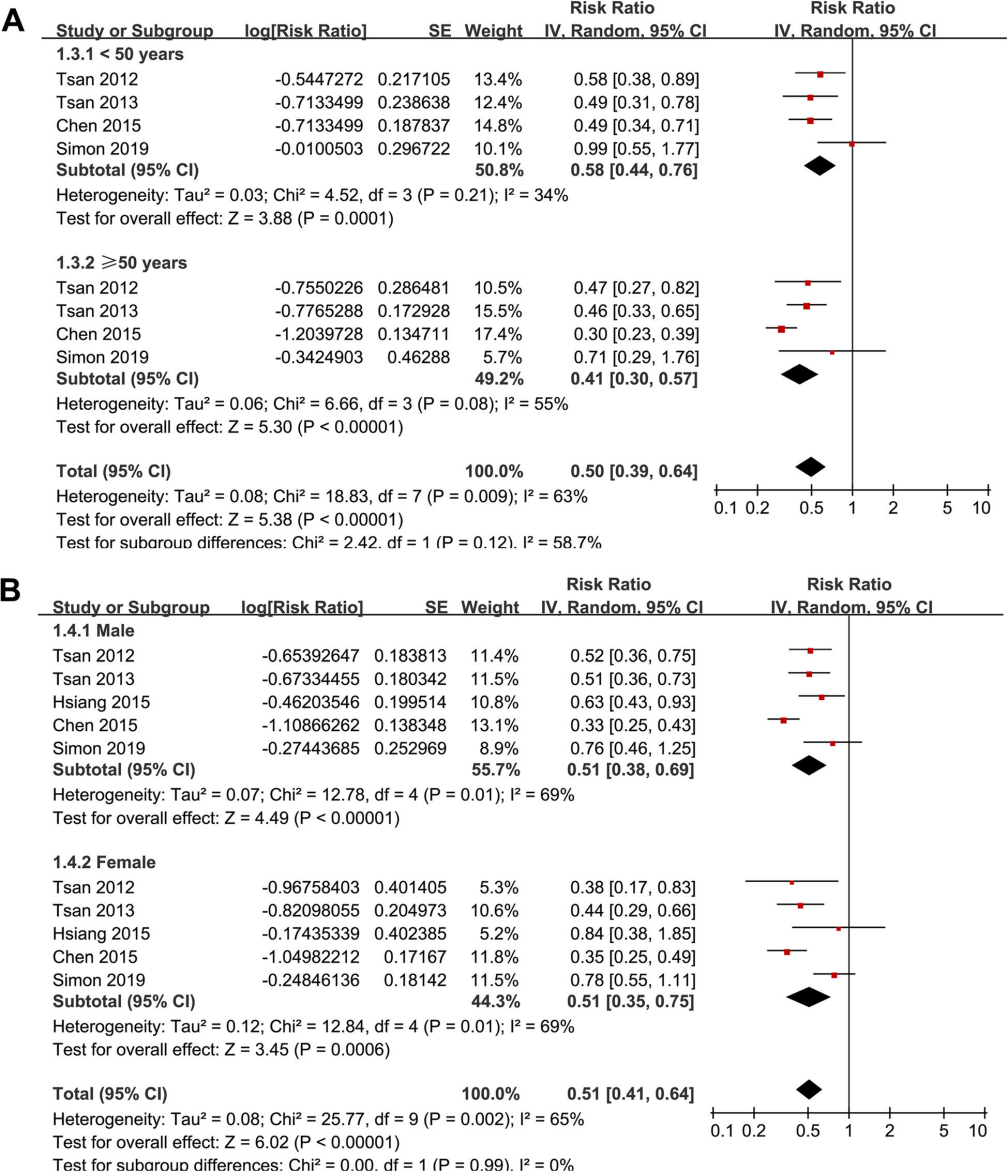
Stratified analyses for the meta-analysis of the association between statin use and HCC risk in patients with HBV or HCV infection; **a** stratified analyses according to patient age; and **b** stratified analyses according to patient sex

**Fig. 4:**
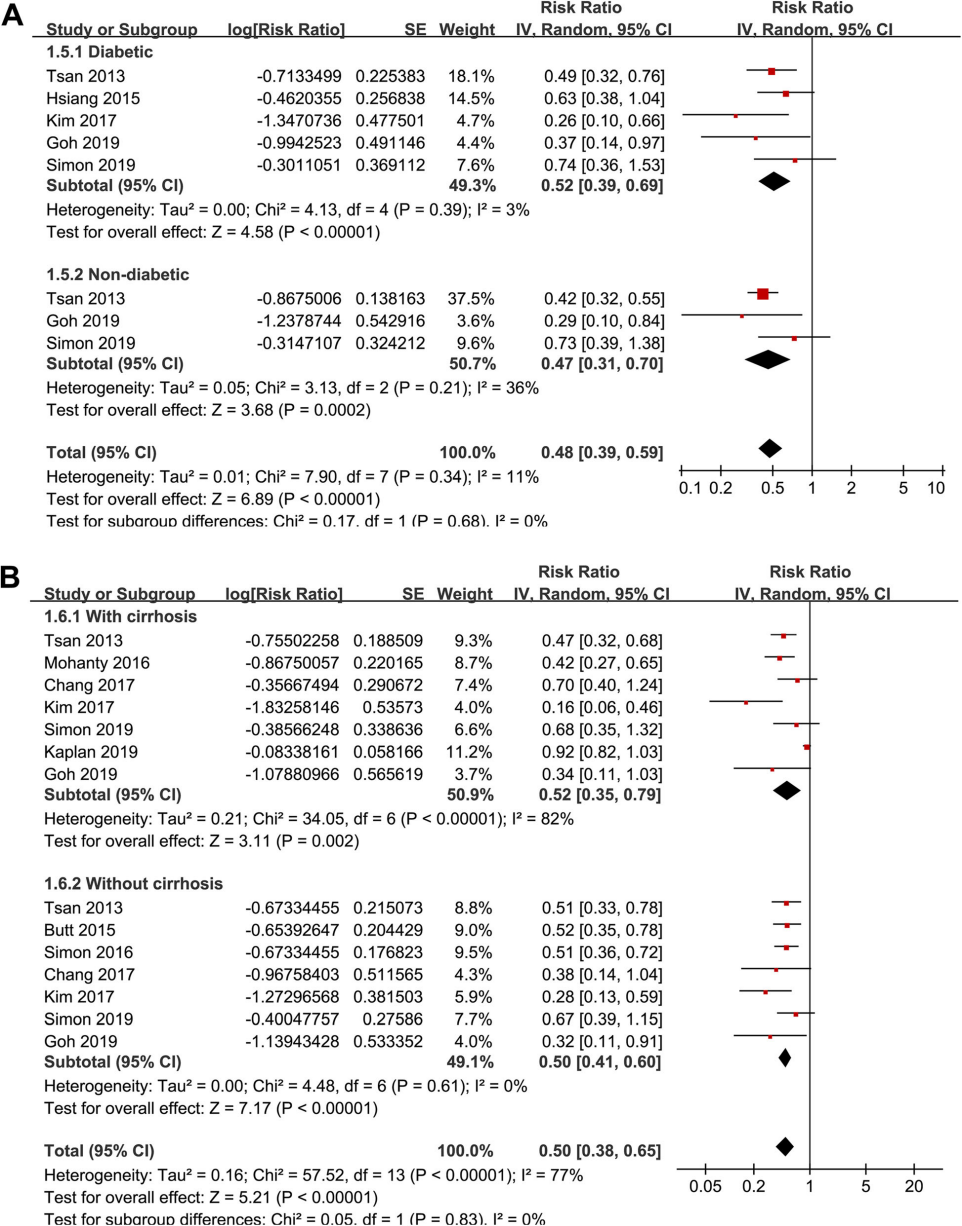
Stratified analyses for the meta-analysis of the association between statin use and HCC risk in patients with HBV or HCV infection; **a** stratified analyses according to diabetic status of patient; and **b** stratified analyses according to with or without cirrhosis

**Fig. 5:**
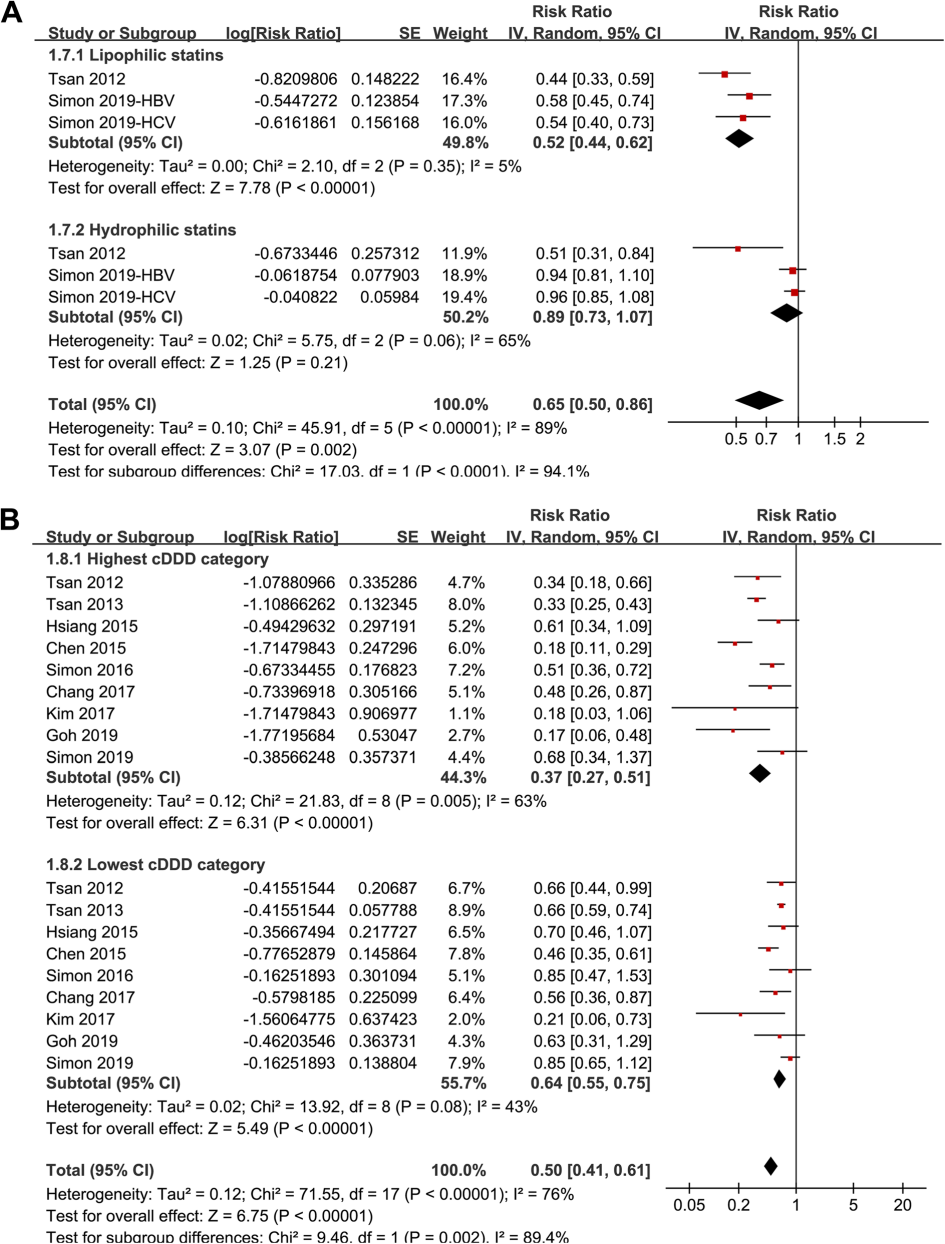
Stratified analyses for the meta-analysis of the association between statin use and HCC risk in patients with HBV or HCV infection; **a** stratified analyses according to the properties of statins (lipophilic or hydrophilic); and **b** stratified analyses according to the accumulative dosages of statins

Stratified analyses showed that the association between statin use and reduced risk of HCC was consistent in patients with HBV (RR: 0.52, 95% CI: 0.40 to 0.68, *p* < 0.001) or HCV infection (RR: 0.58, 95% CI: 0.43 to 0.77, *p* < 0.001; Fig. 2b), in patients < 50 years (RR: 0.58, 95% CI: 0.44 to 0.76, *p* < 0.001) or ≥ 50 years (RR: 0.41, 95% CI: 0.30 to 0.57, p < 0.001; Fig. 3a), in males (RR: 0.51, 95% CI: 0.38 to 0.69, *p* < 0.001) or females (RR: 0.51, 95% CI: 0.35 to 0.75, *p* < 0.001; Fig. 3b), in diabetic (RR: 0.52, 95% CI: 0.39 to 0.69, *p* < 0.001) or non-diabetic patients (RR: 0.47, 95% CI: 0.31 to 0.70, *p* < 0.001; Fig. 4a), and in patients with (RR: 0.52, 95% CI: 0.35 to 0.79, *p* = 0.002) or without cirrhosis (RR: 0.50, 95% CI: 0.41 to 0.60, *p* < 0.001; Fig. 4b). The association between statin use and reduced risk of HCC were not significantly affected by the above patient characteristics (p for subgroup difference all > 0.05). However, stratified analyses with three datasets in each stratum showed that use of lipophilic statins was associated with reduced risk of HCC in patients with HBV or HCV infection (RR: 0.52, 95% CI: 0.44 to 0.62, *p* < 0.001), but not for hydrophilic statins (RR: 0.89, 95% CI: 0.73 to 1.07, *p* = 0.21; p for subgroup difference < 0.001; Fig. 5a). Nine studies reported the potential dose-response relationship between statin use and risk of HCC according to the cDDD of statins [13, 14, 16, 17, 19, 20, 22–24]. However, difference cut-off values for cDDD were used, which prevented a dose-response analysis in our meta-analysis. Subsequently, we performed stratified analyses comparing the association between statin use and HCC risk in patients with highest and lowest cDDD categories in each study. Results showed that the association between statin use and reduced risk of HCC was more remarkable in patients with highest cDDD category for statin prescription (RR: 0.37, 95% CI: 0.27 to 0.51, *p* < 0.001) compared to those with lowest cDDD category (RR: 0.64, 95% CI: 0.55 to 0.75, *p* < 0.001; p for subgroup difference = 0.002; Fig. 5b).

### Publication bias

**Fig. 6:**
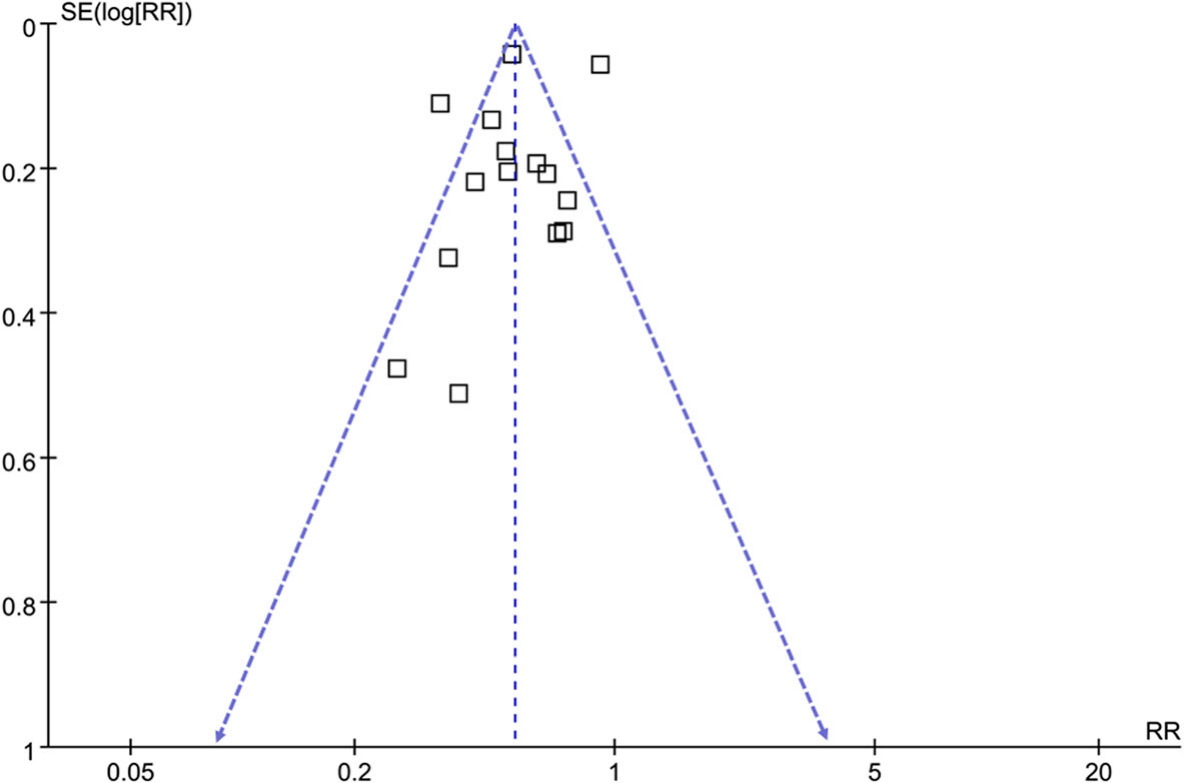
Funnel plots for the publication bias underlying the meta-analysis of the association between statin use and HCC risk in patients with HBV or HCV infection

The funnel plots for the meta-analysis of the association between statin use and HCC risk in patients with HBV or HCV infection were shown in Fig. 6. These plots were symmetry on visual inspection, suggesting low risk of publication bias. Results of Egger’s regression test also suggested low possibility of publication bias (*p* = 0.188).

## Discussion

By summarizing the current evidence from epidemiological studies, our meta-analysis showed that statin use was indepdently associated with reduced risk of HCC in patients with HBV or HCV infection. Subsequently stratified analyses showed that the association between statin use and reduced risk of HCC in these patients were consistent in patients with HBV and HCV infection, in elder (≥ 50 years) and younger (< 50 years) patients, in males and females, in diabetic and non-diabetic, and in those with and without cirrhosis. Moreover, exploring stratified analyses showed that use of lipophilic statins was associated with reduced risk of HCC in patients with HBV or HCV infection, but not for hydrophilic statins. Besides, the association between statin use and reduced risk of HCC in these patients was more remarkable in patients with highest accumulative dose of statin prescription compared to those with lowest accumulative dose category. Taken together, these results demonstrated that statin use was indepdently associated with a reduced risk of HCC in patients with HBV or HCV infection, which may be primarily driven by studies with lipophilic statins and probably via a dose-dependent manner. Although large-scale prospective cohort studies and RCTs are needed to validate these findings, results of this meta-analysis highlight the potential role of statins as chemoprevention agents for HCC in patients with HBV or HCV infection.

To the best of our knowledge, our study is the first meta-analysis focusing on the association between statin use and HCC risk in patients with HBV or HCV infection. The strengths of our study included follows. Firstly, this meta-analysis included only longitudinal follow-up studies, which could therefore establish a sequential association between statin use and reduced risk of HCC in patients with HBV or HCV infection. Secondly, we only studies with adequate adjustment of confounding factors, which therefore may suggest an independently association between statin use and reduced risk of HCC in these patients. Thirdly, we used sensitivity analysis to confirm the robustness of the finding, which was not primarily driven by either of the included study. Finally, multiple stratified analyses were performed to evaluate the stability of the results, which showed that association between statin use and reduced risk of HCC in these patients were consistent and not affected by hepatitis virus type, patient age, sex, diabetic status, and with and without cirrhosis. These results supported the hypothesis that statins may be applied as a chemoprevention agent against the development of HCC in high-risk patients with HBV or HCV infection. Since no RCTs have been published in this field, our results highlighted the need of large-scale RCTs to validate the potential chemoprevention role of statins for HCC.

Results of our stratified analyses showed that use of lipophilic statins was associated with reduced risk of HCC in patients with HBV or HCV infection, but not for hydrophilic statins. However, only three datasets were available for each stratum of the stratified analyses, and the results were mainly driven by one study [22]. Therefore, the results should be interpreted cautiously. Interestingly, previous studies did show that lipophilic statins seem to confer more remarkable anticancer efficacy than hydrophilic statins in some cancers, such as in gynecological cancers expressing high levels of HMG-CoA reductase [32]. The mechanisms for the potential different anticancer efficacies between lipophilic and hydrophilic statins remain to be determined. In addition, we found that the association between statin use and reduced risk of HCC was more remarkable in patients with highest accumulative dose of statin prescription compared to those with lowest accumulative dose category, suggesting a possible dose-dependent manner under the association. However, since the included studies applied cDDD with various cut-off values for categorization of statin dose, large scale studies are warranted to validate the dose-dependent association between statin use and reduced HCC risk in patients with HBV or HCV infection.

The potential molecular mechanisms underlying the chemoprevention effects of statins for HCC may be multiple. An early experimental study showed that combinatorial treatment with statin and protein kinase C-beta inhibitor displayed enhanced anti-tumor efficacy in cultured HCC cells and in a mouse model of HCC [33]. Subsequent studies showed that inhibition of HMG-CoA reductase by atorvastatin blocks both MYC phosphorylation and activation, suppressing tumor initiation and growth in vivo in a transgenic model of MYC-induced HCC as well as in human HCC-derived cell lines [34]. Moreover, in mouse and human HCC cell lines, treatment with fluvastatin, simvastatin, atorvastatin, rosuvastatin or lovastatin are all associated with induced cellular apoptosis in a p53 dependent manner [35]. Modulation other molecular pathways, such as inhibition of signal transducer and activator of transcription 3/SKP2 axis [36], inhibition of SRC/FAK cue [37], and activation of AMPK et al. [38] have also been involved in the potential anti-HCC effects of statins. The key mechanisms underlying the potential anti-HCC efficacy of statins in patients with HBV or HCV infection deserve further investigations.

Our study has limitations Firstly, significant heterogeneity was found for the meta-analysis. Although stratified analyses were performed to evaluate the patient and statin prescription characteristics on the outcome, we could not exclude some other study characteristics that may also contribute to the heterogeneity, such as concurrent medications including antiviral agents [39] and metformin [40]. Both have been indicated to confer anticancer effects. Moreover, due to the limited studies, results of some stratified analyses should be interpreted very cautiously, such as the findings that lipophilic statins and hydrophilic statins may be associated with HCC risk differently. This finding was mainly driven by one include study [22] as previously discussed. In addition, although we included studies with adjusted data, residual factors may remain existing which may confound the association, such as chronic alcoholism [41] and metabolic liver diseases [42]. Finally, a causative association between statin use and reduced HCC risk in patients with HBV or HCV infection could not be derived based on our finding, since this study was a meta-analysis of observational studies. Our finding should be considered as hypothesis-generating. Effect of additional statin therapy on HCC incidence in patients with HBV or HCV infection should be validated in large-scale RCTs.

## Conclusions

In conclusion, results of meta-analysis demonstrated that statin use was indepdently associated with a reduced risk of HCC in patients with HBV or HCV infection, which may be primarily driven by studies with lipophilic statins and probably via a dose-dependent manner. Satins may be potential chemoprevention agents for HCC in patients with HBV or HCV infection.

## Data Availability

All relevant data for this study are presented in tables, figures and supplementary materials.

## Abbreviations

HCC: Hepatocellular carcinoma
HBV: Hepatitis B virus
HCV: Hepatitis C
RR: Risk ratio
HMG-CoA: 3-hydroxy-3-methylglutaryl CoA
MOOSE: Meta-analysis of Observational Studies in Epidemiology
RCT: Randomized controlled trial
CI: Confidence intervals
SE: Stand error
